# Cognitive complaints mediate the influence of sleep disturbance and state anxiety on subjective well-being and ill-being in adult community volunteers: a cross sectional study

**DOI:** 10.1186/s12889-022-12936-0

**Published:** 2022-03-22

**Authors:** Kuniyoshi Toyoshima, Masahiko Ichiki, Takeshi Inoue, Akiyoshi Shimura, Jiro Masuya, Yota Fujimura, Shinji Higashi, Ichiro Kusumi

**Affiliations:** 1 Department of Psychiatry, Hokkaido University Graduate School of Medicine, Kita 15, Nishi 7, Sapporo, 060-8638 Japan; 2 Department of Psychiatry, Tokyo Medical University, Shinjuku-ku, Tokyo, 160-0023 Japan; 3 Department of Psychiatry, Tokyo Medical University Hachioji Medical Center, Tokyo, 193-0998 Japan; 4 Department of Psychiatry, Ibaraki Medical Center, Tokyo Medical University, Ami-machi, Inashiki-gun, Ibaraki, 300-0395 Japan

**Keywords:** Sleep disturbance, State anxiety, Cognitive complaints, Subjective well-being, Subjective ill-being, Mediator

## Abstract

**Background:**

Sleep disturbance, state anxiety, and cognitive complaints (CCs) have been recognized as important issues in public health. Although the mediating role of CCs has been proposed, their role in the relationships between sleep disturbance, state anxiety, and subjective well-being (SWB) and subjective ill-being (SIB) are not yet fully understood. This study used path analyses to investigate whether CCs mediate these relationships.

**Methods:**

The study recruited 523 Japanese adult volunteers using convenience sampling. Participants completed the Pittsburgh Sleep Quality Index, State–Trait Anxiety Inventory (Form Y), Cognitive Complaints in Bipolar Disorder Rating Assessment, and Subjective Well-Being Inventory to evaluate sleep disturbance, state anxiety, CCs, and SWB and SIB, respectively. Path analyses were conducted to assess the mediating effects of CCs.

**Results:**

The path analyses showed significant indirect associations of sleep disturbance and state anxiety with SWB (*p* = 0.024 and *p* = 0.012) and SIB (*p* < 0.001 and *p* < 0.001), respectively, mediated by CCs. Furthermore, there were significant indirect associations of sleep disturbance with CCs (*p* < 0.001), SWB (*p* < 0.001), and SIB (*p* < 0.001), via state anxiety, respectively.

**Conclusions:**

This study suggests that CCs mediate the associations of sleep disturbance and state anxiety with SWB and SIB, respectively, in adult community volunteers. To address SWB and SIB associated with sleep disturbance and state anxiety, evaluating CCs may be useful in public mental health. Our findings will encourage health care workers to assess CCs more systematically. Future studies may need to target CCs to develop interventions for SWB and SIB.

**Supplementary Information:**

The online version contains supplementary material available at 10.1186/s12889-022-12936-0.

## Background

*Health* is a state of complete physical, mental and social well-being and not merely an absence of disease or infirmity [1]. Both subjective well-being (SWB) and subjective ill-being (SIB) influences mental health [2], in which SWB correlates with the cognitive process of contentment [3] and SIB correlates with the negative psychological constructs [4]. Hence, SWB has been defined as positive affect, whereas SIB has been defined as negative affect [2]. However, the absence of SWB does not necessarily lead to SIB, and vice versa [5]. Therefore, important factors that affect mental health should be evaluated in not only SIB but also in SWB [6].

Both sleep disturbance and state anxiety are currently important issues in mental health. Sleep disturbance has been defined as poor quality sleep, and state anxiety has been defined as transient emotional anxiety [7, 8]. A close relationship between sleep disturbance and state anxiety has been shown, and sleep disturbance increases state anxiety [7, 8]. In occupational mental health, cognitive impairments caused by sleep disturbance increase the risk of workplace accidents [9–11]. Not only sleep disturbance but also state anxiety affect cognitive functioning, which in turn influences work performance [12]. Recent research also suggests that reducing anxiety may improve sleep quality and cognitive function [13]. In this context, cognitive impairment is a crucial factor in considering the influences of sleep disturbance and state anxiety on mental health.

*Cognitive complaints* (CCs) are defined as subjective cognitive problems that are perceived in daily life [14]. CCs consist of the following perceived cognitive functions; executive function, processing speed, working memory, verbal learning and memory, attention/concentration, and mental tracking [14]. For example, feeling that it takes longer than usual to complete a daily task suggests subjective impairment in processing speed. Various factors such as neurocognitive dysfunction and depressive symptoms affect CCs [14–16]. Furthermore, CCs influence the quality of life for individuals with psychiatric illnesses as well as for those in the general adult population [17–19]. Thus, CCs have been considered one of the important current issues in mental health [19–21].

The mediating role of CCs has recently been reported in several settings. First, CCs mediate the relations of depressive symptoms on work productivity and life quality [19, 22]. Second, CCs mediate the influence of affective factors on social function [23]. Third, CCs mediate the associations of having the history of childhood maltreatment with adulthood functional disability [24]. Recent studies reported that sleep disturbance and state anxiety directly affected CCs, and CCs directly affected SIB [25, 26]. However, whether CCs mediate the relations of sleep disturbance and state anxiety with SWB and SIB is not yet fully understood. Our study aimed to assess the mediating role of CCs on the relationships among sleep disturbance, state anxiety, SWB and SIB using mediation analyses in our path models. Furthermore, we also aimed to provide key insights into the role of CCs on mental health problems associated with sleep disturbance and state anxiety.

## Methods

### Study participants

The research used convenience sampling to recruit 597 adult volunteers from Tokyo between April 2017 and April 2018. This investigation was part of a larger research, in which some questionnaires were conducted to evaluate the relations of CCs with social functioning in Japanese adults [19].

Study inclusion criteria were: age ≥ 20 years; no current serious physical illness; no organic brain damage; and ability to provide agreement to participate in this investigation. We excluded 74 recruited individuals who did not complete the questionnaire. The final analysis included the data of 523 participants. They were volunteers for this research and completed the questionnaires at home or their workplace. The questionnaires were answered using paper and pen. They took approximately 40–60 min to complete the assessments, which included providing demographic information.

### Measures

#### Demographic characteristics

We assessed the participants’ demographic characteristics using self-administered questionnaires. Psychiatric history was evaluated using the following question: “Do you have any mental illness that you have treated by going to the hospital or taking prescription medications in the past?” Ongoing psychiatric treatment was evaluated using the following question: “Do you have any mental illnesses that you are currently treating, such as by going to the hospital or taking prescription medications?” In the present study, the word “drinking” refers to the consumption of alcohol at least once a month, and the word “smoking” refers to smoking at least one cigarette per day.

#### Sleep disturbance

The Pittsburgh Sleep Quality Index (PSQI) was used to evaluate various aspects of subjective sleep disturbance during the previous month [27]. The index comprises 7 subscales, and each subscale score ranged from 0–3 points. The PSQI global score was obtained by summing all component scores. For instance, the PSQI item 1 was “During the past month, what time have you usually gone to bed at night?” This research used the Japanese version of the PSQI, which was developed after obtaining permission from the original authors [28]. The content and language validity of the PSQI Japanese version has been confirmed [28, 29]. The overall reliability coefficient of the Japanese version was high (Cronbach's alpha = 0.77) [28]. For the PSQI global score in this version, a score of 5.5 was the considered as the cutoff point, which provided 85.7% sensitivity and 86.6% specificity for primary insomnia [29]. This study also used a clinical cutoff point of 5.0, meaning that a PSQI global score > 5 indicated poor quality sleep [30].

#### State anxiety

The State–Trait Anxiety Inventory (Form Y) (STAI-Y) was used to evaluate the severity of state and trait anxiety. The inventory was composed of 40-items using a 4-point scale for each item in the 20-item state anxiety section and the 20-item trait anxiety section [31]. For instance, the STAI-Y item 1 was “I feel calm.” The STAI-Y scores were analyzed separately for state and trait anxiety, with scores for either section ranging from 20 to 80 points. The Japanese version was conducted in this research, the content and language validity of which has been confirmed [32]. In the present study, only the STAI-Y score for state anxiety was used. In the Japanese version, the reliability of state anxiety for male (Cronbach’s alpha = 0.92) and female (Cronbach’s alpha = 0.92) was high, and the mean scores of male (47.3 ± 10.4) and female (45.9 ± 10.2) were also reported [33].

#### CCs

The Cognitive Complaints in Bipolar Disorder Rating Assessment (COBRA) measures CCs that are perceived in daily life [14]. The COBRA evaluates executive function, processing speed, working memory, verbal learning and memory, attention/concentration, and mental tracking. It is composed of 16 items and uses a 4-point scale for each item. For instance, COBRA item 1 was “Do you have difficulty remembering peoples’ names?” The total score was obtained by adding the scores for each item [14]. The Japanese version of COBRA was developed after obtaining permission from the original authors [34]. This research used the COBRA Japanese version, for which the content and language validity has been confirmed [34]. COBRA had one-factor structure [14, 34], and the overall reliability coefficient of the Japanese version was high (Cronbach's alpha = 0.887) [34]. The COBRA total score of > 14 indicates moderate-to-severe CCs [19, 20].

#### SWB and SIB

The Subjective Well-Being Inventory (SUBI) measures SWB and SIB and consists of 2 domains and 40 items—19 items for SWB and 21 items for SIB [2]. The inventory for SWB consisted of 6 subscales and for SIB consisted of 4 subscales. The subscales of SWB are as follows: “general well-being” (positive affect), “expectation-achievement congruence,” “confidence in coping,” “transcendence”, “family group support,” and “social support.” The subscales of SIB are as follows: “inadequate mental mastery,” “perceived ill-health,” “deficiency in social contacts,” and “general well-being” (negative affect). The “primary group concern” subscale is included in both SWB and SIB axes. For instance, the SUBI item 1 was “Life-interesting.” A 3-point Likert scale (ranging from 1 to 3) was used for scoring each item—the higher the score, the better the state in both SWB and SIB. In other words, the higher score of SWB indicates high level of SWB, while the higher score of SIB indicates low level of SIB. This research used the Japanese version, for which the validity and reliability has been confirmed [35, 36]. In the Japanese version, the reliability of SWB (Cronbach's alpha = 0.89) and SIB (Cronbach's alpha = 0.86) were high [36]. In the present study, 19 items for SWB and 21 items for SIB (SWB scores range from 21 to 57 and SIB scores range from 28 to 63) were used for statistical analysis. In the Japanese version, the scores below 31 for SWB and 43 for SIB indicate bad conditions for SWB and SIB, respectively [36].

### Statistical analysis

Pearson correlation analysis was conducted using Bonferroni adjustment to investigate the associations between PSQI global score (sleep disturbance), STAI-Y state anxiety score (state anxiety), COBRA total score (CCs), and the SUBI scores for both SWB and SIB. Two multiple regression analyses were conducted with the forced-entry method as follows: the dependent factors were SUBI SWB and SIB scores; the independent factors were sociodemographic characteristics, PSQI score, STAI-Y state anxiety score, and COBRA total score. Before conducting multiple regression analyses, linearity was verified using a normal probability plot. Path analyses were performed to investigate the relations of sleep disturbance, state anxiety, and CCs with SWB and SIB. Because of the saturation model, the study did not refer to the goodness-of-fit index. Our path model was a saturation model; hence, the sample size was calculated to be a minimum of 100 [37, 38]. The standardized path coefficients indicated the strengths of direct, indirect, and total effects. For statistical analysis, the study used STATA/MP 16 (StataCorp, College Station, TX, USA), except for the path analysis, which was conducted using Mplus version 8.4 (Stata Corp). *p* < 0.05 was considered to be statistically significant.

## Results

The sociodemographic characteristics and clinical assessments are presented in Table 1. The number of study participants with a PSQI score > 5 was 246 (47.04%), which indicated that they had poor sleep quality, and the number of study participants with a COBRA total score > 14 was 88 (16.83%), which indicated that they had moderate to severe CCs. Notably, the proportion of participants with poor sleep quality was higher than that in a previous study in Japan [30].

**Table 1:** Basic findings (*N* = 523)

Demographic characteristics	Mean (SD)	Range of variables (Minimum–Maximum)	N (%)
Age	40.88 (11.82)	20–77	
Sex (Male/ Female)			233/290 (44.55/55.45)
Married			346 (66.16)
Education (years)	14.70 (1.80)	9–21	
Current employment			514 (98.28)
Psychiatric history			53 (10.13)
Ongoing psychiatric treatment			19 (3.63)
Drinking			341 (65.20)
Smoking			99 (18.93)
**Clinical assessments**	**Mean (SD)**	**Range of variables (Minimum–Maximum)**	**N (%)**
Sleep disturbance	5.73 (3.51)	0–21	
State anxiety	41.14 (9.63)	20–71	
CCs	8.20 (6.45)	0–32	
SWB	38.47 (6.83)	21–57	
SIB	51.53 (6.40)	28–63	

*CCs* Cognitive complaints, *SWB* Subjective well-being, *SIB* Subjective ill-being *SD* Standard deviation

The results of Pearson correlation analysis are presented in Additional file 1. Sleep disturbance was positively related with state anxiety and CCs, whereas sleep disturbance was negatively correlated with high level of SWB and low level of SIB. State anxiety was positively related with CCs, whereas state anxiety was negatively related with high level of SWB and low level of SIB. CCs were significantly and negatively related with high level of SWB and low level of SIB. High level of SWB was significantly and positively correlated with low level of SIB.

### Multiple regression analysis

Significant positive predictors of high level of SWB were female sex, married status, years of education, and undergoing current psychiatric treatment (Table 2). Significant negative predictors of high level of SWB were age, sleep disturbance, state anxiety, and CCs. Significant positive predictors of low level of SIB were age and married status. Significant negative predictors of low level of SIB were having psychiatric history, sleep disturbance, state anxiety, and CCs.

**Table 2:** Multiple regression analyses (*N* = 523)

	**SWB** F (12, 510) = 25.41, *p* < .0001		**SIB** F (12, 510) = 38.63, *p* < .0001	
**Independent factors**	***β***	***VIF***	***β***	***VIF***
Age	− .14^**^	1.39	.14^***^	1.39
Sex: 1; Male, 2; Female	.12^**^	1.26	.01	1.26
Married status: 1; No, 2; Yes	.16^***^	1.17	.07^*^	1.17
Years of education	.22^***^	1.44	.06	1.44
Currently employed, 1; No, 2; Yes	− .02	1.07	− .01	1.07
Psychiatric history, 1; No, 2; Yes	-.04	1.32	− .14^***^	1.32
Current psychiatric treatment, 1; No, 2; Yes	.10^*^	1.28	.04	1.28
Drinking, 1; No, 2; Yes	.04	1.17	.04	1.17
Smoking, 1; No, 2; Yes	.03	1.11	− .00	1.11
Sleep disturbance	− .13^**^	1.28	− .15^***^	1.28
State anxiety	− .37^***^	1.29	− .39^***^	1.29
CCs	− .11^**^	1.17	− .27^***^	1.17
**Adjusted *R***^***2***^	**0.36**		**0.46**	

*CCs* Cognitive complaints, *SWB* Subjective well-being, *SIB* Subjective ill-being, *β* Standardized regression coefficients, *VIF* Variance inflation factor

^*^p < 0.05, ^**^p < 0.01, ^***^p < 0.001

### Path analysis of SWB

**Fig. 1:**
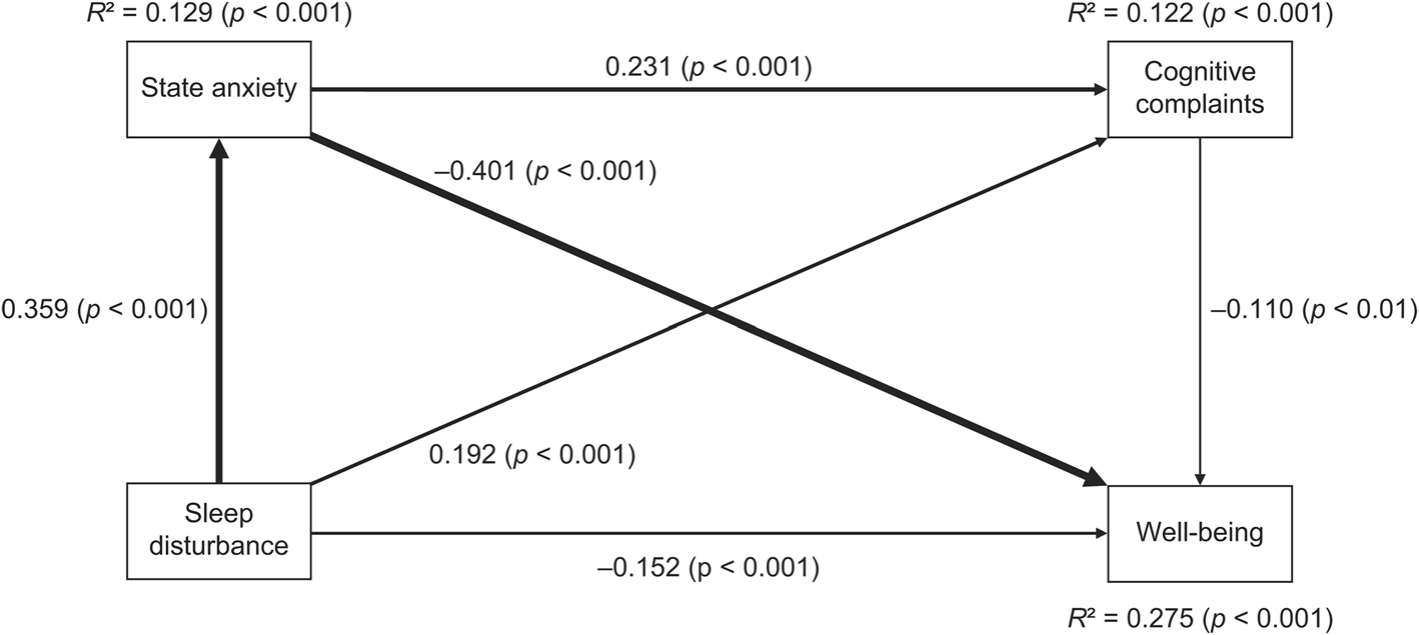
Relations among sleep disturbance, state anxiety, cognitive complaints, and well-being. The value beside the arrow represents the direct standardized path coefficient. The widths of lines show the strength of direct effects

The associations among sleep disturbance, state anxiety, CCs, and SWB are presented in Table 3. The *R*^*2*^ of SWB was 0.275, meaning that the model explained 27.5% of variability in SWB, and all the paths were statistically significant (Fig. 1).

**Table 3:** Path analysis of SWB (*N* = 523)

Direct effect to			
From	state anxiety	CCs	SWB
sleep disturbance	0.359^***^	0.192^***^	− 0.152^***^
state anxiety		0.231^***^	− 0.401^***^
CCs			− 0.110^**^
**Indirect effect to**			
	state anxiety	CCs	SWB
sleep disturbance		0.083^***^ (via state anxiety)	− 0.144^***^ (via state anxiety)
			− 0.009^*^ (via state anxiety and CCs)
			− 0.021^*^ (via CCs)
state anxiety			− 0.025^*^ (via CCs)
**Total indirect effect to**			
sleep disturbance		0.083^***^	− 0.174^***^
state anxiety			− 0.025^*^
**Total effect to**			
	state anxiety	CCs	SWB
sleep disturbance	0.359^***^	0.275^***^	− 0.326^***^
state anxiety		0.231^***^	− 0.426^***^
CCs			− 0.110^**^

^*^p < 0.05, ^**^p < 0.01, ^***^p < 0.001

Regarding direct effects, sleep disturbance predicted state anxiety, CCs, and SWB. State anxiety predicted CCs and SWB. Further, CCs predicted SWB. Regarding indirect effects, sleep disturbance predicted CCs via state anxiety. Sleep disturbance also predicted SWB via state anxiety, via CCs, and via both state anxiety and CCs. State anxiety predicted SWB via CCs. Therefore, the mediating roles of state anxiety and CCs were shown in the model.

### Path analysis of SIB

**Fig. 2:**
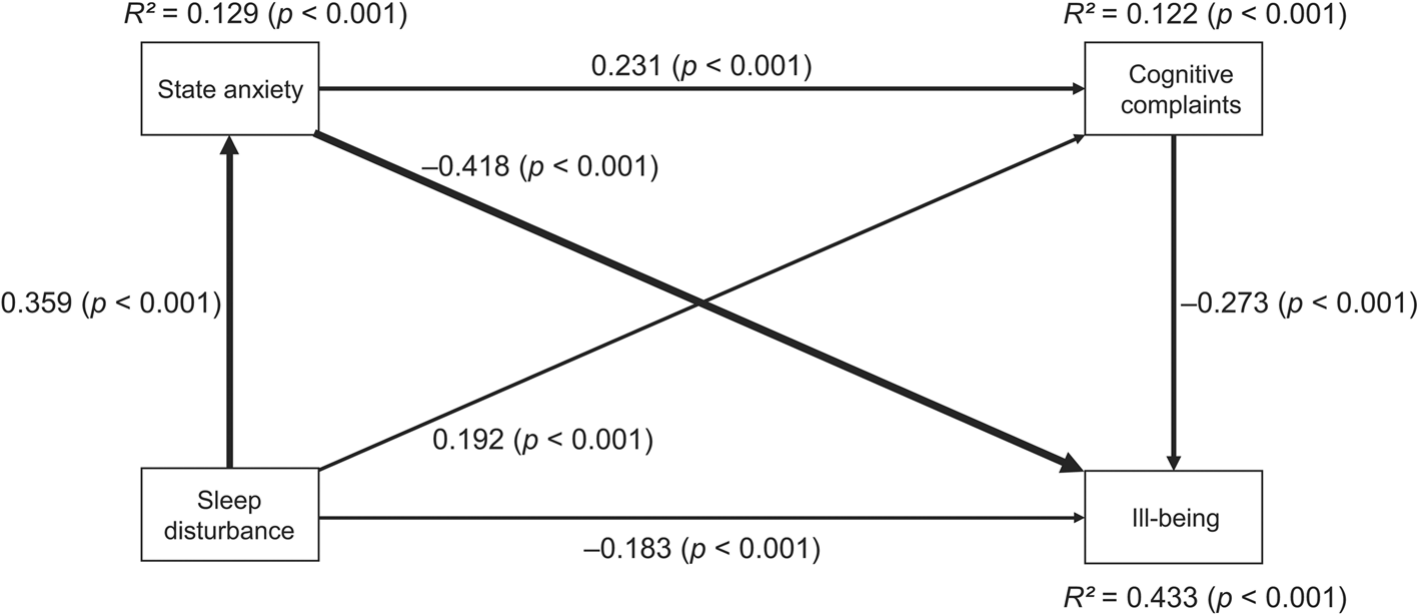
Relations among sleep disturbance, state anxiety, cognitive complaints, and ill-being. The value beside the arrow represents the direct standardized path coefficient. The widths of lines show the strength of direct effects

The associations among sleep disturbance, state anxiety, CCs, and SIB are presented in Table 4. The *R*^*2*^ of SIB was 0.433, meaning that the model explained 43.3% of variability in SIB, and all the paths were statistically significant (Fig. 2).

**Table 4:** Path analysis of SIB (*N* = 523)

Direct effect to			
From	state anxiety	CCs	SIB
sleep disturbance	0.359^***^	0.192^***^	− 0.183^***^
state anxiety		0.231^***^	− 0.418^***^
CCs			− 0.273^***^
**Indirect effect to**			
	state anxiety	CCs	SIB
sleep disturbance		0.083^***^ (via state anxiety)	− 0.150^***^ (via state anxiety)
			− 0.023^**^ (via state anxiety and CCs)
			-0.052^***^ (via CCs)
state anxiety			-0.063^***^ (via CCs)
**Total indirect effect to**			
sleep disturbance		0.083^***^	− 0.225^***^
state anxiety			− 0.063^***^
**Total effect to**			
	state anxiety	CCs	SIB
sleep disturbance	0.359^***^	0.275^***^	− 0.408^***^
state anxiety		0.231^***^	− 0.481^***^
CCs			− 0.273^***^

^*^p < 0.05, ^**^p < 0.01, ^***^p < 0.001

Regarding direct effects, sleep disturbance predicted state anxiety, CCs, and SIB. State anxiety predicted CCs and SIB, and CCs predicted SIB. Regarding indirect effects, sleep disturbance predicted CCs via state anxiety. Sleep disturbance predicted SIB via state anxiety, via CCs, and via both state anxiety and CCs. State anxiety predicted SIB via CCs. Consequently, the mediating roles of state anxiety and CCs were shown in the model.

## Discussion

The present study suggests that CCs mediate the relations of sleep disturbance and state anxiety with SWB and SIB. Consistent with the previous study in Japan, both sleep disturbance and state anxiety directly affected CCs in our path models [25]. Nevertheless, to the authors’ best knowledge, these mediating roles of CCs are new findings.

A recent research reported the associations among sleep quality, anxiety, and neurocognitive function in a U.S. sample, including various races (e.g., Caucasian; 52.8%, Asian; 1.7%), in which both sleep disturbance and anxiety were found to decrease neurocognitive function [13]. Our results confirm these findings and suggest that sleep disturbance and anxiety are not only related to decreased performance on neurocognitive tests, but also predict subjective cognitive problems. In the future, the mediating role of cognitive function (as measured by neurocognitive tests) in the relationship between sleep disturbance, anxiety, and CCs in a Japanese sample needs to be investigated.

Our findings suggest the importance of assessing CCs for public health. In Japan, CCs have been gradually noted in mental health because subjective cognitive problems are detected in daily living and affect psychological and social problems [19]. However, the assessment of CCs in general adults has not yet been fully established in public health. By using a brief instrument to evaluate CCs, such as COBRA, health care workers can assess CCs more systematically. Our results indicate that it may be important to assess sleep quality and mental health, particularly anxiety, among individuals who report CCs. Addressing the underlying sleep and mental health concerns may help reduce subjective cognitive problems. A recent study suggested that internet-delivered computerized cognitive behavioral therapy may improve sleep disturbance and related symptoms, including anxiety, as well as the quality of life of individuals with insomnia in Japan [39]. Considering our findings, CCs could mediate the effectiveness of online cognitive behavioral therapy for improving the quality of life of individuals with sleep disturbance and anxiety. Thus, in the future, assessing CCs may help elucidate the mechanism of action of interventions. Currently, online cognitive behavioral therapy should be applied with care because the cost effectiveness and applicability to a given population is not yet fully investigated in Japan [40]. In considering the intervention strategy to minimize CCs, the area of cognitive dysfunction and the personal lifestyle may need to be considered together. Namely, simultaneous interventions for both cognitive dysfunction and environmental adjustments may be useful. The present study may provide useful insights into the development of intervention strategies for CCs among the Japanese population.

Regarding the characteristics of our sample, notably, most of the participants were employed at the time of the assessment. In a previous Japanese study, CCs mediated the influence of insomnia and anxiety on loss of work productivity [25]. Additionally, a recent study suggested that the loss of work productivity mediates the influence of CCs on SWB and SIB in Japanese adult workers [41]. Therefore, sleep disturbance and anxiety might lead to poorer CCs, and CCs might decrease work productivity, and consequently, SWB and SIB might be exacerbated in Japanese workers. In future studies, the role of CCs in the relationship of sleep disturbance and anxiety with work productivity, SWB, and SIB in Japanese workers needs to be investigated.

In terms of sleep disturbance, the participants of this study tended to report poor sleep quality compared with that in a previous study in Japan [30]. The present research included some individuals with ongoing psychiatric treatment, which may also affect sleep quality. Previous studies suggested that sleep disturbance endangers the health and safety of workers, and insomnia is the most important predictor of work accidents [42–44]. Occupational stress and violence also affect sleep disturbance of workers [45, 46]. With regard to the geographic location, living in the urban area was correlated with poor sleep in prior studies in Japan [47, 48]. In this study, all participants were recruited in urban areas, which could have influenced the participants’ sleep quality. In the future, working and living environments need to be assessed in more detail to develop health promotion activities for individuals with sleep disturbance.

Finally, it is important to discuss the mediating role of CCs on SWB and SIB. Previous studies in Japan reported the mediating roles of CCs in the relationship between depressive symptoms and work productivity, between affective factors and social function, and between having history of childhood maltreatment and functional disability [22, 23, 24]. Furthermore, affective factors mediated the relationship of having history of childhood abuse with SWB and SIB in a Japanese sample [6]. Hence, the influence of affective factors may be as important as that of CCs when evaluating individuals' quality of life, including SWB and SIB. Regarding the relationship of affective factors with CCs in Japanese population, depressive symptoms have been found to mediate the relationship of affective temperaments (cyclothymic, depressive, irritable, and anxious temperament) with CCs [49]. To the best of our knowledge, the mediating role of CCs in the relationship of affective factors with SWB and SIB is yet to be fully understood. Therefore, in future studies, the roles of CCs and affective variables in well-being should be explored in more detail.

### Limitations

Regarding data collection, this study excluded 74 recruited individuals who did not complete the questionnaire. Another limitation in this study is that the sample potentially included some individuals with untreated and clinically undiagnosed psychiatric symptoms. All study subjects were recruited from a community in Japan; consequently, the results could not be generalized to other communities and countries. All participants were adults aged ≥ 20 years; thus, the results may not be generalized to children and adolescents. Regarding the factors that affect CCs, childhood maltreatment, affective temperaments, and depressive symptoms were previously established [23, 24]; hence, these factors may affect the mediating role of CCs. In addition, CCs are perceived in daily life [14], hence, they are susceptible to psychosocial factors. However, the path models in the current study could not evaluate the influence of psychosocial functioning on CCs, which could be a study limitation. Further, the volunteers were not asked about their cognitive status-MCI, dementia, etc. Also, the absence of objective cognitive assessment could be a limitation of this study. The test–retest reliability analysis of the questionnaire was not done in this study. The definition of "smoking" lacked the information about timeline, which could be a study limitation. We included some individuals with ongoing psychiatric treatment, which may also affect sleep quality. Finally, a cross-sectional survey research could not determine causality among the parameters.

## Conclusion

To address SWB and SIB as related to sleep disturbance and state anxiety, assessing CCs may be useful for public mental health. Our findings point to the importance of assessing CCs more systematically by the health care workers. Future studies may need to develop interventions that target CCs to ameliorate SWB and alleviate SIB.

## Supplementary Information

**Additional file 1.** Pearson Correlation Analysis Using Bonferroni Adjustment (*N* = 523).

## Data Availability

The datasets used and/or analyzed during the current study are available from the corresponding author on reasonable request.

## Abbreviations

CCs: Cognitive complaints
COBRA: Cognitive Complaints in Bipolar Disorder Rating Assessment
PSQI: Pittsburgh Sleep Quality Index
*R*^*2*^: Coefficient of determination
SD: Standard deviation
SIB: Subjective ill-being
SUBI: Subjective Well-Being Inventory
SWB: Subjective well-being
VIF: Variance inflation factor
