# Supplementary material for: Cognitive complaints mediate the influence of sleep disturbance and state anxiety on subjective well-being and ill-being in adult community volunteers: a cross sectional study
Source: BMC Public Health. 2022 Mar 22;22:566. doi: 10.1186/s12889-022-12936-0 (PMC8939175; doi:10.1186/s12889-022-12936-0)
Supplement: Supplementary file 1 — Additional file 1. Pearson Correlation Analysis Using Bonferroni Adjustment (N = 523). [file 12889_2022_12936_MOESM1_ESM.docx]

**Additional file 1.** Pearson Correlation Analysis Using Bonferroni Adjustment (*N* = 523)

|  | PSQI | STAI-Y state anxiety | COBRA | SUBI well-being |
| --- | --- | --- | --- | --- |
| PSQI | - |  |  |  |
| STAI-Y state anxiety | .36^*^ | - |  |  |
| COBRA | .28^*^ | .30^*^ | - |  |
| SUBI well-being | −.33^*^ | −.49^*^ | −.27^*^ | − |
| SUBI ill-being | −.41^*^ | −.57^*^ | −.45^*^ | .47^*^ |

^*^*p* < 0.001.

COBRA, Cognitive Complaints in Bipolar Disorder Rating Assessment; PSQI, Pittsburgh Sleep Quality Index; STAI-Y, State–Trait Anxiety Inventory Form Y; SUBI, Subjective Well-Being Inventory.
